# Cerebral Autoregulation in Patients Treated With V-Vecmo For Severe Ards

**DOI:** 10.1186/2197-425X-3-S1-A509

**Published:** 2015-10-01

**Authors:** V Fanelli, AT Mazzeo, I Battaglini, S Caccia, M Boffini, D Ricci, S El Qarra, G Izzo, R Urbino, PP Terragni, L Brazzi, M Rinaldi, VM Ranieri, L Mascia

**Affiliations:** University of Turin, Anesthesia and Intensive Care, Turin, Italy; University of Turin, Cardiac Surgery, Turin, Italy; University of Rome 'La Sapienza', Anesthesia and Intensive Care, Rome, Italy

## Introduction

In patients with severe ARDS refractory to conventional mechanical ventilation, venous-venous extracorporeal membrane oxygenation (v-vECMO) is a rescue therapy able to restore normal values of PaO2 and PaCO2, maintaining the lung at rest. Hypoxemia and hypercapnia occurring in patients with severe ARDS may negatively affect cerebral autoregulation.

## Objectives

The hypothesis of the present study was that, in patients with severe ARDS, impaired cerebral autoregulation due to gas exchange derangement, may be restored by application of v-vECMO.

## Methods

Clinical prospective observational study. Inclusion criteria: severe ARDS (P/F ≤ 100), requiring v-vECMO. Exclusion criteria: previous diagnosed neurological diseases. the following parameters were recorded before (pre) and after institution of v-vECMO (post): mean arterial pressure (MAP), cerebral blood flow velocity in middle cerebral artery (MCA FV) by transcranial Doppler, and alveolar gas exchange (PaCO2, PaO2). Cerebral autoregulation was assessed by Pearson linear regression coefficient (Mx index) between MCA FV and MAP during spontaneous fluctuations of MAP (cut-off of Mx ≥ 0.2 was adopted to define impaired autoregulation).

## Results

Five severe ARDS patients were enrolled. Three female with mean age of 40.2 ± 16.5, APACHE II 33 ± 6.4, SAPS II 60.4 ± 21.85, secondary to influenza H1N1 pneumonia (n = 3), cystic fibrosis (n = 1), pleural empyema (n = 1), duration of v-vECMO of 16 days (range 2-46). See Table 1.

**Table 1:** *Physiological data before and after ECMO.*

Patient Code	MCA Flow Velocity (cm/sec) - PRE	MCA Flow Velocity (cm/sec) - POST	Mx - PRE	Mx - POST	PaO2 (mmHg) - PRE	PaO2 (mmHg) - POST	PaCO2 (mmHg) - PRE	PaCO2 (mmHg) - POST
1	128	62	0.57	0.2	58	91	115	32
2	74	52	0.59	0.27	53	158	42	36
3	100	42	0.35	0.19	60	75	88	36
4	54	57	0.25	0.71	63	75	69	34
5	130	72	0.14	-0.11	78	220	84	52

Patient 4 evolved to brain death on day 1 and was then excluded.

Paired t-test was used for analysis. MAP was 82 ± 9 and 94 ± 17, pre and post ECMO respectively. Mx significantly changed from 0.41 ± 0.2 to 0.13 ± 0.16 (p = 0.009); both PaO2 and PaCO2 significantly improved (p = 0.05).

## Conclusions

In patients with severe ARDS our preliminary data suggest that v-vECMO is able to restore cerebral autoregulation that is impaired because of severe gas exchange derangement.

